# Depth Edge Filtering Using Parameterized Structured Light Imaging

**DOI:** 10.3390/s17040758

**Published:** 2017-04-03

**Authors:** Ziqi Zheng, Seho Bae, Juneho Yi

**Affiliations:** College of Information and Communication Engineering, Sungkyunkwan University, Suwon 16419, Korea; zqseria@skku.edu (Z.Z.); bseho@skku.edu (S.B.)

**Keywords:** depth edge filter, depth detection, structured light

## Abstract

This research features parameterized depth edge detection using structured light imaging that exploits a single color stripes pattern and an associated binary stripes pattern. By parameterized depth edge detection, we refer to the detection of all depth edges in a given range of distances with depth difference greater or equal to a specific value. While previous research has not properly dealt with shadow regions, which result in double edges, we effectively remove shadow regions using statistical learning through effective identification of color stripes in the structured light images. We also provide a much simpler control of involved parameters. We have compared the depth edge filtering performance of our method with that of the state-of-the-art method and depth edge detection from the Kinect depth map. Experimental results clearly show that our method finds the desired depth edges most correctly while the other methods cannot.

## 1. Introduction

**Figure 1:**
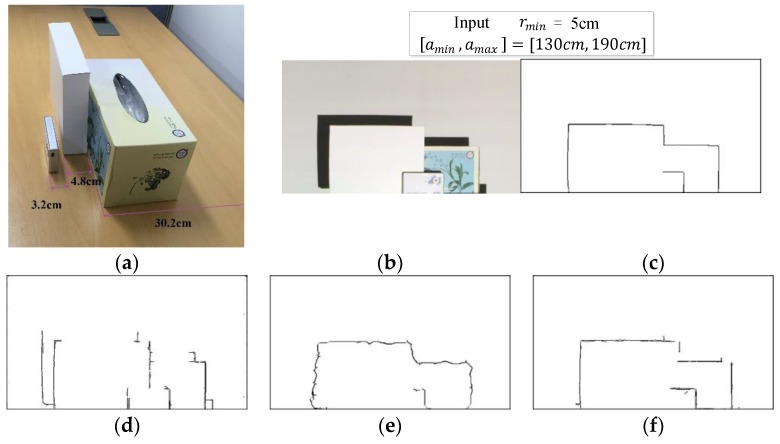
Depth edges, which have the minimum depth difference of 5 cm in the specified range of distances [130 cm, 190 cm] from the projector/camera, are detected in (**a**). The camera’s view is shown in (**b**). Depth edge filtering results are presented for (**c**) ground truth; (**d**) method in [8]; (**e**) Kinect, and (**f**) our method.

The goal of this work, as illustrated in Figure 1, is to accurately find all depth edges that have the minimum depth difference, $r_{min}$, in the specified range of distances, $\left[a_{min},\;a_{max}\right],$ from the camera/projector. We call this “depth edge filtering”. We propose a structured light based framework that employs a single color pattern of vertical stripes with an associated binary pattern. Due to the accurate control of the parameters involved, the proposed method can be employed for applications where detection of object shape is essential—for example, when a robot manipulator needs to grasp an unknown object by identifying inner parts with a certain depth difference.

There has been a considerable amount of research on structured light imaging in the literature [1,2,3,4,5]. Here, we only mention some recent works to note. Barone et al. [1] presented a coded structured light technique for surface reconstruction using a small set of stripe patterns. They coded stripes in De Bruijn sequence and decomposed color stripes to binary stripes so that they can take advantage of using a monochromatic camera. Ramirez et al. provided a method [2] to extract correspondences of static objects through structured light projection based on De Bruijn sequence. To improve the quality of depth map, Shen et al. presented a scenario [3] for depth completion and denoising. Most works have aimed at surface reconstruction and there have been a few works for the purpose of depth edge filtering. One notable technique was presented to create a depth edge map for nonphotorealistic rendering [6]. They capture a sequence of images in which different light sources illuminate the scene from various positions. Then, they use shadows in each image to assemble a depth edge map. However, this technique is incapable of the control of parameters such as range of distances from the camera/projector and depth difference.

A while ago, in [7,8], similar control of parameterizing structured light imaging was presented. They employed structured light with a pattern comprising black and white horizontal stripes of equal width, and detected depth edges with depth difference $r\geq r_{min}$ in a specified range of distances. Since the exact amount of pattern offset along depth discontinuities in the captured image can be related to the depth value from the camera, they detected depth edges by finding detectable pattern offset through thresholding of Gabor amplitude. They automatically computed the width of stripe by relating it with the amount of pattern offset.

A major drawback of the previous methods is that they did not address the issue of shadow region. Regions that the projector light cannot reach create shadow regions and result in double edges. Figure 1d shows the result of the method in [8] for the given parameters where, in the shadow regions, double edges and missing edges appear. Furthermore, due to the use of simple black and white stripes, the exact amount pattern offset may not be measurable depending on the object location from the camera. This deficiency requires additionally employing several structured lights with width of stripe doubled, tripled, etc.

**Figure 2:**
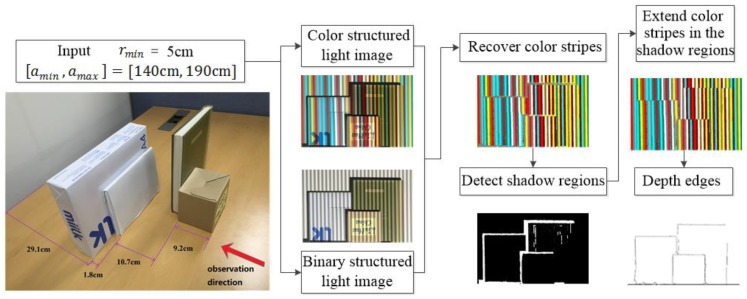
An overview of our method.

In this work, we present an accurate control of depth edge filtering by overcoming the disadvantages of the previous works [7,8]. We provide an overview of our method in Figure 2. We opt to use a single color pattern of vertical stripes with an associated binary pattern as shown in Section 3. The use of the binary pattern helps with recovering the original color of the color stripes accurately. We give the details in Section 3. Given the input parameters, $\left[a_{min},\;\;a_{max}\right]$ and $r_{min}$, stripe width, w, is automatically computed to create the structured light patterns necessary to detect depth edges having depth difference greater or equal to $r_{min}$. We capture structured light images by projecting the structured light patterns on the scene. We first recover the original color of the color stripes in the structured light images in order not to be affected by the textures on the scene. Then, for each region of homogeneous color, we use a Support Vector Machine (SVM) classifier to decide whether a given region is from shadow or not. After that, we obtain color stripes pattern images by filling in shadow regions using the color stripes that otherwise have been projected there. We finally apply Gabor filtering to the pattern images to produce the depth edges with depth difference greater or equal to $r_{min}$.

We have compared the depth edge filtering performance of our method with that of [8] and the Kinect sensor. Experimental results clearly show that our method finds the desired depth edges most correctly while the other methods cannot. The main contribution of our work lies in an accurate control of depth edge filtering using a novel method of effective identification of color stripes and shadow removal in the structured light image.

## 2. Parameterized Structured Light Imaging 

By parameterized structured light imaging, we refer to the technologies using structured light imaging that can control associated parameters. To the best of our knowledge, Park et al.’s work [7] was the first of its kind. In our case, the controlled parameters are the minimum depth difference, $r_{min}$, a target range of distances, $\left[a_{min},\;\;a_{max}\right]$, and the width of stripe, w. The basic idea in [7] to detect depth edges is to exploit pattern offset along depth discontinuities. To detect depth discontinuities, they consecutively project a white light and structured light onto the scene and extract a binary pattern image by differencing the white light and structured light images. This differencing effectively removes texture edges. After removal of texture edges, they basically detected the locations where pattern offset occurs to produce depth edges. In contrast, we achieve the effect of texture edge removal by recovering the original color stripes in the color structured image. Details will be given in the next section.

**Figure 3:**
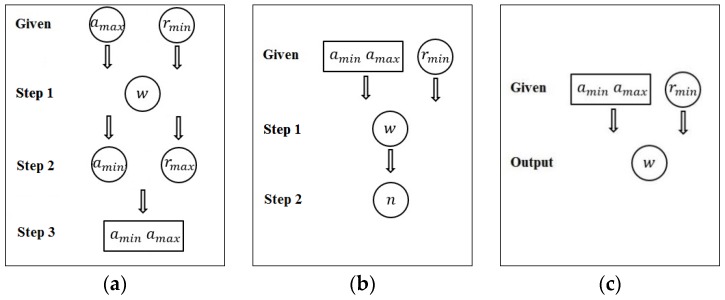
Control of key parameters: (**a**) method in [7]; (**b**) method in [8]; (**c**) our method.

The control of parameters developed in [7] can be seen in Figure 3a where $a_{max}$ and $r_{min}$ are given as the input parameters; then, the width, w, and $a_{min}$ are determined. However, it was awkward that $a_{min}$ is found at a later step from other parameters. A substantial improvement over this method was made in [8] so that $\left[a_{min},\;\;a_{max}\right]$ and $r_{min}$ are given as the input parameters. Given the input parameters, the method provides the width of stripes, w, and number of structured light images, n as shown in Figure 3b. They also showed its application to the detection of silhouette information for visual hull reconstruction [9]. In our work, we achieve much simpler control of the key parameters by employing a color pattern as can be seen in Figure 3c. While the methods in [7,8] need several structured light images, we use a single color pattern and an associated binary pattern.

**Figure 4:**
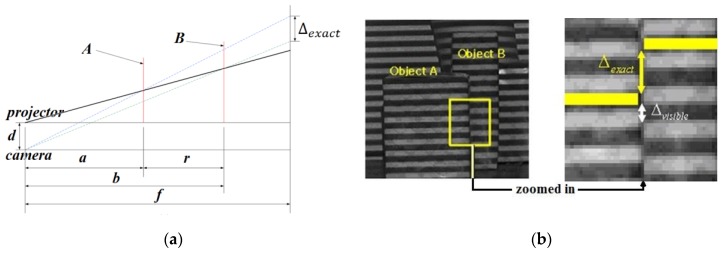
Imaging geometry and pattern offset: (**a**) side view of image geometry; (**b**) $\Delta_{exact}$ vs. $\Delta_{visible}$.

To better describe our method, let us revisit the key Equation (1) for the modelled imaging geometry of a camera, projector and object in [7,8]. This Equation can easily be derived from the geometry in Figure 4 using similar triangles:

(1) $$\Delta_{exact}=fd\left(\frac{1}{a}-\frac{1}{b}\right)=\frac{fdr}{a\left(a+r\right)}.$$

Here, a, b and f are the distances of object locations A and B from the projector/camera and virtual image plane from the camera, respectively. $\Delta_{exact}$ denotes the exact amount of pattern offset when the depth difference of object locations A and B is r.

**Figure 5:**
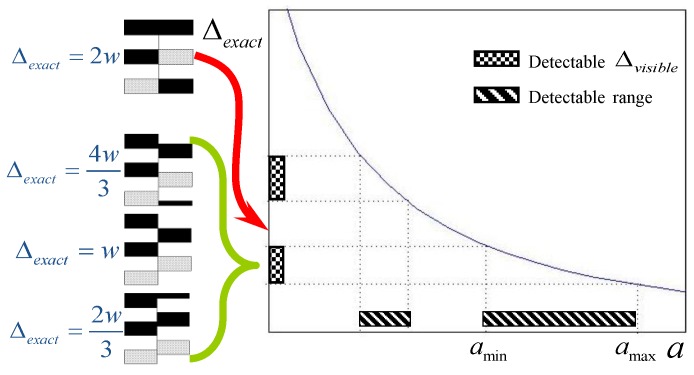
Pattern offset $\Delta_{exact}$ vs. detectable range $\left[a_{min},\;\;a_{max}\right]$.

Since, in [7,8], they used simple black and white stripes with equal width, $\Delta_{exact}$ may not be measurable depending on the object location from the camera. The observable amount of pattern offset, $\Delta_{visible}$, is periodic as the distance of object location from the camera is increased or decreased. With *r* and *d* fixed, the relation between $\Delta_{exact}$ and a depicts that there are ranges of distances where detection of depth edges is difficult due to the lack of visible offset even though $\Delta_{exact}$ is significant. Refer to Figure 5. They have set the minimum amount of pattern offset that is needed to reliably detect depth edges to 2w/3. In order to extend the detectable range, additional structured lights with width of stripe 2w, 4w, etc. are employed to fill the gap of $\Delta_{exact}$ in Figure 5, and the corresponding range, a, of object locations is extended. In contrast, because we use color stripes pattern, $\Delta_{exact}$ is equivalent to $\Delta_{visible}$. Thus, there is no need to employ several pattern images.

## 3. Use of Color Stripes Pattern

We opt to use color stripe patterns by which we can extend the range of distances by filling in the gap of $\Delta_{exact}$ in Figure 5. We consider a discrete spatial multiplexing method as a proper choice [10] because it shows negligible errors and only a simple matching algorithm is needed. We employ four colors: red, cyan, yellow and white. We also make use of two versions for each color: bright and dark. That is, their RGB (Red, Green and Blue) values are [*L*,0,0], [0,*L*,*L*], [*L*,*L*,0], and [*L*,*L*,*L*], *L* = 255 or 128, where *L* denotes lightness intensity. To create a color pattern, we exploit De Brujin sequences [11] of length 3, that is, any sequence of three color stripes is unique in a neighborhood. This property helps identify each stripe in the image captured by the camera.

**Figure 6:**
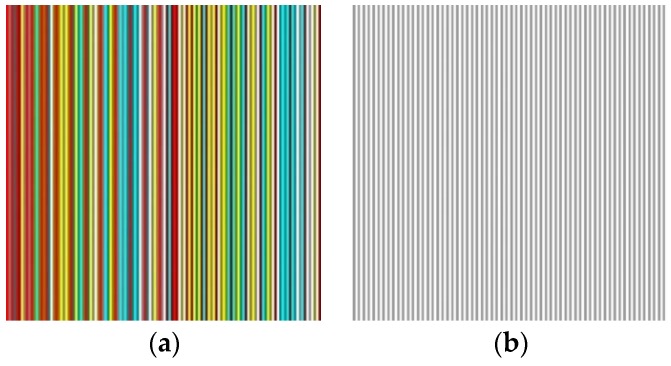
Color stripes pattern with an associated binary pattern: (**a**) color pattern; (**b**) binary pattern.

Additionally, we use an associated binary stripes of which RGB values can be represented as [*L*,*L*,*L*], *L* = 255 or 128. That is, we also make use of bright (*L* = 255) and dark (*L* = 128) versions for binary stipes. We have designed the stripe patterns so that in both color stripes and binary stripes, bright and dark stripes appear alternately. The color stripes are associated with the binary stripes so that bright stripes in the color pattern correspond to dark stripes in the binary pattern. Refer to Figure 6. This setting indeed greatly facilitates the solution when recovering the original color of color stripes in the color structured light image by referencing the lightness of binary stripes in the binary structured light image.

The most attractive advantage of employing color stripes pattern of De Bruijin sequence is that $\Delta_{exact}$ is the same as the amount of visible pattern offset $\Delta_{visible}$. We can safely set the minimum amount of pattern offset necessary for detecting depth edges to w/2. In addition, 2w/3 was used in [7,8]. Thus, the width of stripe width, w, is computed using Equation (2) [8]:

(2) $$w=\frac{2fdr_{min}}{\left(a_{max}+r_{min}\right)\left(a_{max}+2r_{min}\right)}.$$

## 4. Recovery of the Original Color of Stripes

**Figure 7:**
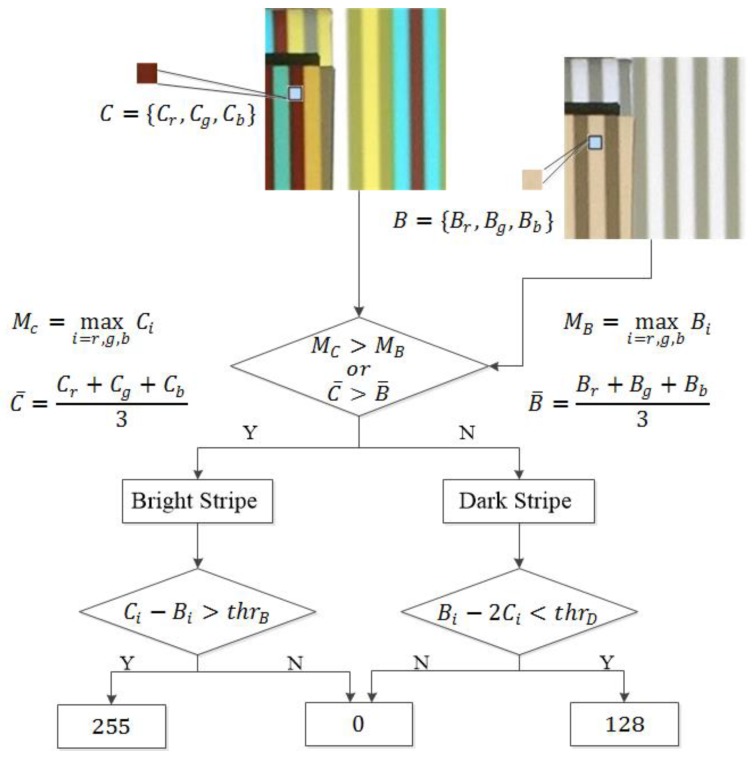
Flow of recovering the original color of color stripes.

The problem of recovering the original color of color stripes in the structured light image is to determine the lightness *L* in each color channel. We exploit the associated binary image as reference to avoid decision errors. Figure 7 shows the procedure of recovering the original color of color stripes. The procedure consists of two steps. For every pixel in the color structured image, we first decide whether it comes from a bright (*L* = 256) color stripe or dark (*L* = 128) color stripe. Then, we recover the value of *L* in each color channel. 

Let us denote a pixel in the color structured light image and its corresponding pixel in the binary structure light images, *C* and *B*, respectively. $C_{i}$ and $B_{i}$, i=r,g,b, represent their RGB values. Since bright stripes in the color pattern correspond to dark stripes in the binary pattern, it is very likely that a pixel from a bright color stripe appears brighter than its corresponding pixel from the binary stripe when they are projected onto the scene. Thus, in most cases, we can make a correct decision simply by comparing the max value of $C_{i}$ with the max value of $B_{i}$, i=r,g,b. However, since RGB values of stripes in the captured images are affected by the object surface color, we may have decision errors, especially for pixels on the object surface that have high values in one channel. For example, when object surface color is pure blue [0,0,255] and color stripe is bright red [255,0,0], the RGB values of a pixel on the object surface in the color and binary structured images can appear as [200,5,200] and [100,100,205], respectively. In this case, only comparison of max channel value gives a wrong answer. Hence, we employ an additional criterion that compares the average value of all three channels. Through numerous experiments, we have confirmed that this simple scheme achieves correct pixel classification into bright or dark ones.

Next, we decide the value of each channel, *L*. Luminance and ambient reflected light can vary in different situations. We take an adaptive thresholding scheme to make a decision. In case of a pixel in bright color stripes, $C_{i}\in \left\{0,\;255\right\}$ and $B_{i}=128.$ We decide that if $C_{i}-B_{i}>thr_{B},$ then $C_{i}=\;255$; otherwise, $C_{i}=0.$ $thr_{B}$ is determined as Equation (3) and s is computed from training samples:

(3) $$thr_{B}=s\cdot \underset{i=r,g,b}{\max}\left\{C_{i}-B_{i}\right\}.$$

In the case of a pixel in dark color stripes, $C_{i}\in \left\{0,\;128\right\}$ and $B_{i}\;=\;255.$ We decide that if $B_{i}-2C_{i}<thr_{D},$ then $C_{i}=128.$ Otherwise, $C_{i}=0.$ $thr_{D}$ is computed as Equation (4), and b and t are estimated from training samples:

(4) $$thr_{D}=-2b+t\cdot \underset{i=r,g,b}{\min}\left\{B_{i}-2C_{i}\right\}.$$

We set a bias b to ensure that most of the time $thr_{D}$ is positive. This is necessary to deal with any positive $\left\{B_{i}-2C_{i}\right\}$ close to $\min\left\{B_{i}-2C_{i}\right\}$ when $\min\left\{B_{i}-2C_{i}\right\}$ is negative.

The relationship between the original color and captured color is nonlinear. We seek to use a simple statistical method to determine parameters, s, b and t. We collect a series of images. Each set is comprised of three images, $M_{b},$ $M_{g}$ and $M_{w}$, that are captured by projecting black [0,0,0], gray [128,128,128] and white [255,255,255] lights, respectively. $M_{b}$, $M_{g}$ and $M_{w}$ can be viewed as three image matrices that experimentally simulate the observed black, gray and white color. Note that we took every image in the same ambient environment. Usually, the more training samples we collect, the more representative parameters we can get. However, hundreds of samples are sufficient for our estimation in practice. We use multifarious objects in different shapes and with various textures to build scenes. We estimate s as follows:

(5) $$s=\frac{1}{N}\underset{i}{\sum }\min\left\{\frac{\mathrm{minimum}\left(M_{wi}-M_{gi}\right)}{\mathrm{maximum}\left(M_{wi}-M_{gi}\right)}\right\}.$$

*N* is the number of sets, minimum(·) and maximum(·) are element-wise functions and i means the *i*th set. In bright stripes, we already know that $C_{i}-B_{i}$ should be 127 when the channel value is assigned 255 in patterns. Equation (5) is a sampling process about the relationship between the maximum and minimum of $C_{i}-B_{i}$ when projected on the scene. $thr_{B}$ gives the smallest $C_{i}-B_{i}$. If $C_{i}-B_{i}$ is greater than $thr_{B}$ in any channel, we like to believe its value is 255.

We initially model $thr_{D}$ as $t\cdot \underset{i=r,g,b}{\min}\left\{B_{i}-2C_{i}\right\}$ as in Equation (6). This model shares the idea behind Equation (3). It makes $t\cdot \min\left\{B_{i}-2C_{i}\right\}$ become the smallest value that $\min\left\{B_{i}-2C_{i}\right\}$ could be. However in dark stripes, $B_{i}-2C_{i}$ is close to zero. Simply scaling does not affect its sign, which might lead to an inappropriate decision. In order to alleviate external interference, we slightly adjust the threshold model above according to $\min\left\{B_{i}-2C_{i}\right\}$. We increase the threshold when $\min\left\{B_{i}-2C_{i}\right\}$ is rather large or decrease it otherwise. Since $M_{b}$, $M_{g}$ and $M_{w}$ are observed lightness values of *L* = 0, 128 and 255, respectively, $M_{gi}^{\prime }=\frac{M_{wi}-M_{bi}}{2}$ is an estimation of $M_{gi}$. Thus, we adjust the threshold value based on the difference between $M_{gi}$ and $M_{gi}^{\prime }$. Hence, we approximate b as in Equation (7). The threshold for dark stripes is altered slightly in terms of the sign of $\min\left\{B_{i}-2C_{i}\right\}$. s=0.65, t=0.47 and b=50 were used in our experiments:

(6) $$t=\frac{1}{N}\underset{i}{\sum }\frac{\mathrm{minimum}\left(\left|M_{wi}-2M_{gi}\right|\right)}{\mathrm{mean}\left(\left|M_{wi}-2M_{gi}\right|\right)},$$

(7) $$b=\frac{1}{N}\underset{i}{\sum }\max\left\{M_{gi}-M_{gi}^{\prime }\right\}.$$

**Figure 8:**
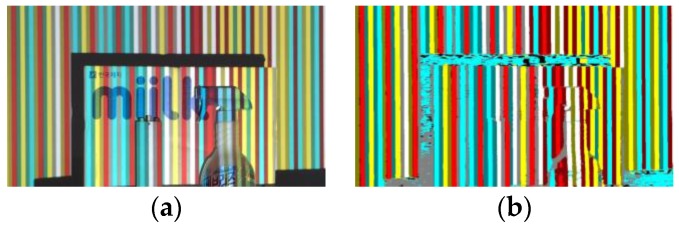
Recovery of the original color of color stripes: (**a**) image captured by the camera; (**b**) recovered color stripes.

Lastly, we check whether the recovered color is one of the four colors we adopt to use. If not, we change it to the most probable color in four. We achieve this in two steps: (1) compare recovered color with four default colors to see how many channels match; (2) among the colors having the most matching channels, choose the color with minimum threshold difference over mismatching channels. Figure 8 shows an experimental result on the recovery of the original color of color stripes. In Figure 8b, the gray and green areas in the lower part and the noisy areas around main objects correspond to shadow regions. Because shadow regions are colorless, color assignment is meaningless. As previously stated, we can ignore texture edges on object surfaces by considering the original color of color stripes.

Although empirically determined parameters are used, the whole thing works pretty well in non-shadow regions. However, recovered color is meaningless in shadow regions where stripe patterns are totally lost. We detect shadow regions and extend color stripes there that otherwise would have been projected. Details follow in the next section.

## 5. Removal of Shadow Regions

In structured light imaging, shadows are created in regions that the projector light cannot reach. In shadow regions, stripe patterns are totally lost, and parameter controlled depth edge detection is not possible there. In order to prevent double edges and missing edges in shadow regions, we proactively identify shadow regions and extend color stripes that otherwise have been projected.

There has been research on natural shadow removal [12,13]. Although their works do not deal with the exactly same scene as ours, some conclusions are valuable. When a region becomes shaded, it becomes darker and less textured. It indicates that colors and textures are important tools to detect shadow regions. After we have recovered the original color of the projected stripes, we divide a recovered color image into simply connected regions of homogeneous color and make a region-based decision whether a given region is from shadow or not. We employ the following features.

### 5.1. Color Feature

We convert recovered color into Lab space and build color histogram. As provided by Guo et al. [12], we set 21 bins in each channel. All the histograms are normalized by the region area. Eliminating parts of non-shadow regions by thresholding of the *L* channel beforehand saves a great deal of time on training and clustering data.

### 5.2. Texture Feature

Textons, a concept stated in [10], can help us build a texture histogram. They construct a series of filters that are derived from a normal 2D Gaussian filter. We apply their filter bank to a large number of experimental images and categorize the data using a k-means algorithm to form k clusters whose mean points are called textons. Every pixel is clustered around its closest texton. Texture histograms are also normalized by the region area.

### 5.3. Angle Feature

Shadow is colorless. We look at each pixel in a color pattern image and its corresponding pixel in a binary pattern image in the RGB space. Let us denote them by C and B, respectively. We form two vectors, $\vec{OC}$ and $\vec{OB}$, from the origin to C and B. The angle between $\vec{OB}$ and $\vec{OC}$ should be small for a pixel in shadow. Shadow probability can be estimated using the cosine value of this angle; however, this angle feature alone is not enough to correctly classify shadow regions.

### 5.4. Classifier Training

**Figure 9:**
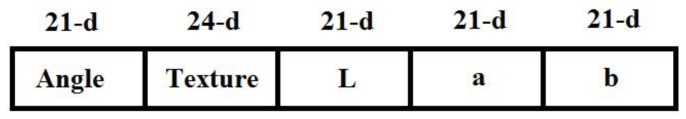
Feature vector used to identify shadow regions.

**Figure 10:**
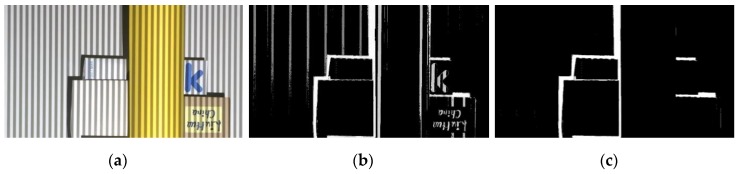
Detection of shadow regions: (**a**) camera’s view of the scene; (**b**) cosine value of angle between $\vec{OB}$ and $\vec{OC}$; (**c**) shadow regions detected using the feature vector in Figure 9.

We use color, texture, and angle features together as in Figure 9 to train an SVM classifier. We number all four colors with two different lightness values so that every pixel is marked as an integer between 1 and 8. We could easily segment the recovered color stripe regions into scraps and cluster it into shadow or non-shadow regions. Each scrap is a training example. We sampled roughly 3000 examples in our experiments. Because the camera is on the upper left side of the projector in our experiments, the shadows must be caused by the left or the top side of objects. This prior helps learn where to find shadow regions in which we extend stripes. Figure 10 shows that shadow regions are accurately detected using our method. Generally, we fill each shadow region with the region above it. As for those shadow regions on the top position, we choose the regions on the right side to replace them.

## 6. Depth Edge Detection

**Figure 11:**
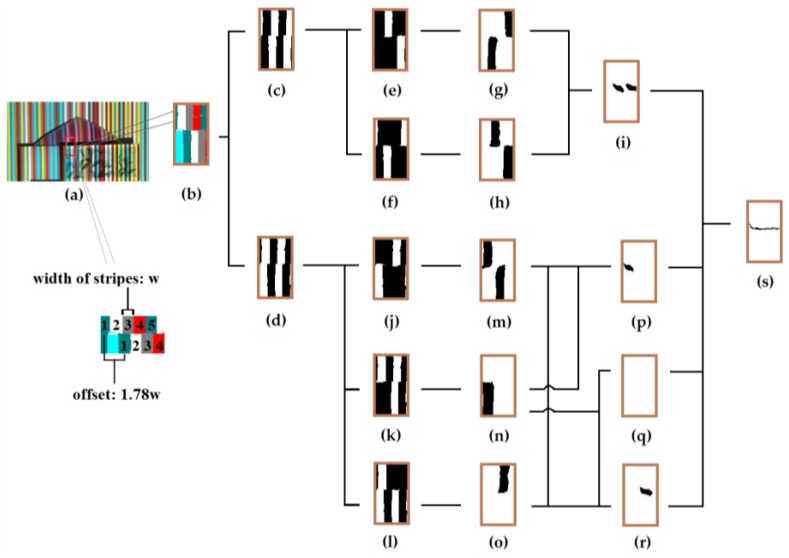
The process of detecting depth edges from the recovered color stripes in a shadow region: (**a**) color stripe pattern image; (**b**) recovered color stripes in the shadow region (**a**); (**c**) binary pattern for dark stripes; (**d**) binary pattern for bright stripes; (**e**) partial pattern without dark cyan stripes; (**f**) partial pattern without gray stripes; (**g**) Gabor response from (**d**); (**h**) Gabor response from (**e**); (**i**) depth edges between dark cyan and gray stripes; (**j**) partial pattern without white stripes; (**k**) partial pattern without cyan stripes; (**l**) partial pattern without red stripes; (**m**) Gabor response from (**j**); (**n**) Gabor response from (**k**); (**o**) Gabor response from (**l**); (**p**) depth edges between cyan and white stripes; (**q**) depth edges between red and cyan stripes; (**r**) depth edges between red and white stripes; and (**s**) depth edges detected as the union of (**i**,**p**,**q**,**r**).

We use Gabor filtering for depth edge detection as in [7,8]. They applied Gabor filtering to black and white stripe patterns to find where the spatial frequency of the stripe pattern breaks. Because depth edge detection using Gabor filtering can only be applied to a binary pattern, we consider bright stripes patterns and dark stripes patterns separately to create binary patterns as can be seen in Figure 11c,d, respectively. Along the depth edge in Figure 11, the upper stripe is supposed to have a different color from the lower one. Then, we exploit color information to detect potential depth edge locations by applying Gabor filtering to binary pattern images where binary stripes for each color are deleted in turn. Note that, as long as there are pattern offsets in the original color pattern image, the amount of offset in the binary patterns, which are obtained by deleting the binary stripe for each color, becomes larger than the original offset amount. This makes the response of Gabor filtering more vivid to changes in periodic patterns. Similar to the previous work [7], we additionally make use of texture edges to improve localization of depth edges. In order to get texture edges, we synthesize a gray scale image of the scene without stripe patterns simply by averaging max channel value of color pattern image and binary pattern image for each pixel. Figure 11 illustrates the process of detecting depth edges of which the offset amount is 1.78w. A Gabor filter of size 2w × 2w is used. Figure 11e,f shows the pattern without dark cyan and gray stripes, respectively. Figure 11g,h is their responses of Gabor filter which have been binarized. The regions of low Gabor amplitude, shown in black, indicate locations of potential depth edges. We process the bright stripes pattern in the same way. Figure 11i,p,q,r includes all the possible combinations of colors along the edges. Thus, the union of them yields depth edges. We simply apply thinning operation to the result of the union in order to get the skeleton.

## 7. Experimental Results

**Figure 12:**
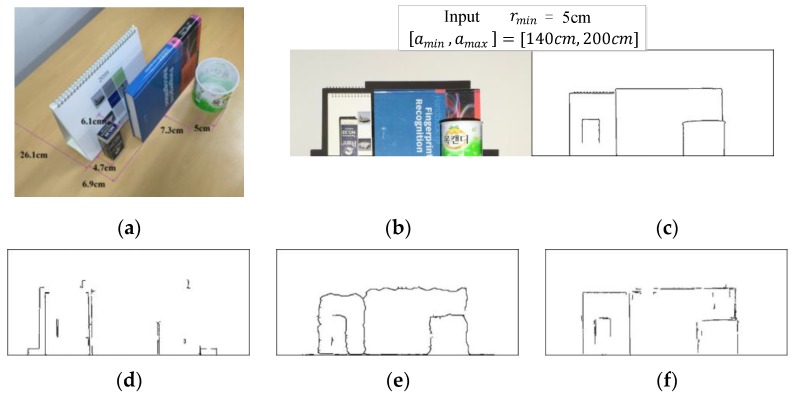
Depth edge filtering results for r≥5 cm: (**a**) experimental setup; (**b**) front view; (**c**) ground truth; (**d**) method in [8]; (**e**) Kinect; (**f**) our method.

We have coded our method in Matlab (2015b, MathWorks, Natick, MA, USA) and the codes have not been optimized. We have used 2.9 GHz Intel Core i5 CPU, 8GB 1867 MHz DDR3 memory (Santa Clara, CA, USA) and Intel Graphics ( Iris Graphics 6100, Santa Clara, CA, USA). Figure 12 shows an example of experimental results. We have compared the performance of our method with those of the previous method [8] and using the Kinect sensor. To produce depth edges from a Kinect depth map, for every pixel, we scan depth values in its circular region of radius 5, and output the pixel if any pixel within its circle has depth difference of $r\geq r_{min}$. The result clearly shows that our method finds the depth edges most correctly for the given parameters while the other methods cannot. Figure 12e shows the result of depth edge detection from the Kinect depth map where straight depth edge segments are not detected as straight. This is because depth values provided by the Kinect sensor along depth edges are not accurate due to interpolation. Irrespective of false positives or false negatives, there are two main causes: inaccurate color recovery and stripes will result in false edges. However, color recovery errors are well contained because we check on the De Bruijn constraint when identifying the original color of stripes. When a shadow region is not detected, false positives occur. On the other hand, when some non-shadow regions are treated as shadow near boundaries, incorrect depth edges are produced. Table 1 lists computation time for each step of our method shown in Figure 2.

**Figure 13:**
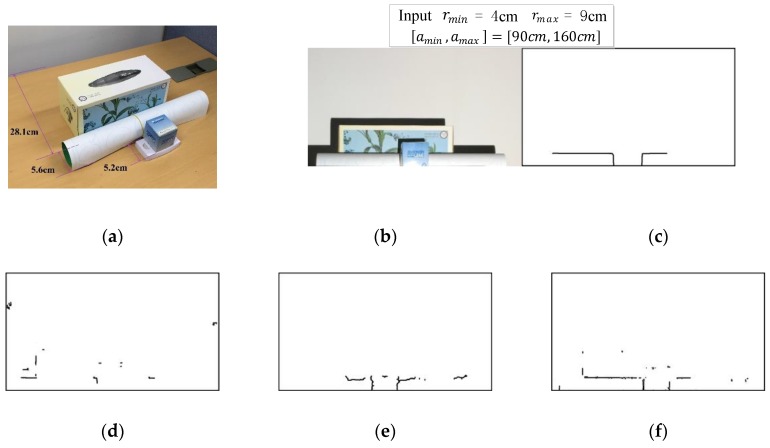
Depth band-pass filtering results for 4 cm ≤r≤ 9 cm: (**a**) experimental setup; (**b**) front view; (**c**) ground truth; (**d**) method in [8]; (**e**) Kinect; (**f**) our method.

Figure 13 depicts an additional experimental result where we find depth edges that satisfy the depth constraint of $r_{min}\leq r\leq r_{max}$. We can achieve this by two consecutive applications of Gabor filtering to the pattern images: The first and second Gabor filter yield depth edges with $r\geq r_{min}$ and $r\geq r_{max}$, respectively, and we remove the depth edges with $r\geq r_{max}$. We can see that our method outperforms the others. While we have provided raw experimental results without any postprocessing operations, the result could be easily enhanced by employing simple using morphological operations.

## 8. Conclusions

We have presented a novel method that accurately controls depth edge filtering of the input scene using a color stripes pattern. For accuracy, we employed an associated binary pattern. Our method can be used for active sensing of specific 3D information about the scene. We think that if a task is to find accurate depth edges, our method provides a better solution. Further research is in progress to use the proposed method to create a sketch of 3D reconstruction by compiling depth edges with various depth differences.

## Figures and Tables

**Table 1:** Computation time.

Image Size	1280 × 800	720 × 576
Recover color stripes	54.35 s	39.50 s
Detect shadow regions	4.10 s	2.12 s
Extend color stripes in the shadow regions	4.94 s	3.45 s
Depth edges detection	5.87 s	3.99 s
Total	69.26 s	49.06 s
